# 

**Published:** 1888-07-21

**Authors:** 


### CONTENTS.

**Provident and Improvident Hospitals** - 253

**The Surgery of the Past** - 254

**The Dead Emperor** - 255

**The British Nurses' Association.** By H.R.H. PRINCESS CHRISTIAN and MR. BURDETT - 256

**Hints for the Sick-room.** By M. M. BASIL, M.A., M.B., Author of "The Commoner Accidents to Life and Limb."—Leeches, Cupping, and Blisters - 257

**The Puddingless Patients** - 258

**New Remedies and Appliances** - 259

**Notes and News** - 260

**Everybody's Page** - 262

**Annotations.**—The Dorset County Asylum —The Massacre of the Innocents.—An Apostle of Long Life - 263

**Within the Wards.**—Flat Feet—Paralysis after Diphtheria—Diarrhœa Treated by Salol—The Bacillus of Cancer - 264

**An Ishmaelite.** By LESLIE KEITH, Author of "The Chilcotes," "East and West," etc. - 265

**Turning Over New Leaves.**—Amputations Treated Antiseptically.—The Science and Art of Surgery - 267

**Amusements with Profit for all Ages.**—For Men and Wo nen.—Children's Corner.—Puzzles, etc. - 268

**EXTRA SUPPLEMENT.—THE NURSING MIRROR.**

En Passant.—A Country Conversation.—Nursing the Insane.—The Slums of Manchester.—Ealing Cottage Hospital.—A Woman's Paradise.—A Brave Nurse - lxi
The Life of a Private Nurse.—The Ipswich Nurses' Home - lxii
A District Matron's Diary - lxiii
Everybody's Opinion.—Nursing for the Middle Classes.—The Want Supplied - lxiii
Nurses for Nervous and Mental Disorders.—Notes and Queries.—The Nurses' Bookshelf.—"Domville's Manual."—Vacancies.—Exchange and Mart - lxiv

